# Construction and Validation of a Gastric Cancer Diagnostic Model based on Blood Groups and Tumor Markers

**DOI:** 10.7150/jca.88190

**Published:** 2024-01-01

**Authors:** Yuhuan Liu, Shasha Chen, Weina Shen, Xiaodong Qu, Songbo Li, Yongquan Shi

**Affiliations:** 1 Xi'an Medical University, Xi'an 710021, Shaanxi Province, China.; 2 State Key Laboratory of Holistic Integrative Management of Gastrointestinal Cancers and National Clinical Research Center for Digestive Diseases, Xijing Hospital of Digestive Diseases, Fourth Military Medical University, Xi'an 710032, China.

**Keywords:** Stomach neoplasms, Tumor markers, ABO blood-group System, Diagnostic prediction model, Receiver operating curve

## Abstract

**Objective:** The aim of this study is to explore the value of combined detection of ABO blood group and tumor markers in the diagnosis of gastric cancer.

**Methods:** A total of 3650 gastric cancer patients treated in our center from January 2015 to December 2019, and 5822 controls were recruited, and divided into training set and validation set according to 7:3. The diagnostic and predictive model of gastric cancer was constructed by binary logistic regression method in the training set. The diagnostic value of the prediction model for gastric cancer was evaluated by calculating the prediction probability P value and drawing the Receiver operating characteristic (ROC) curve, and was verified in the validation set.

**Results:** The Area under the curve (AUC) of the diagnosis and prediction model in the training set was 0.936 (95%CI: 0.926-0.941), the sensitivity was 81.66%, and the specificity was 98.61%. In the validation set, the AUC was 0.941 (95%CI: 0.932-0.950), the sensitivity was 82.33%, and the specificity was 99.02%. Furthermore, the diagnostic model obtained in this study had a high diagnostic value for early gastric cancer patients in the healthy population (AUC of training set, validation set and total population were 0.906, 0.920 and 0.908, respectively).

**Conclusions:** We constructed a diagnostic model for gastric cancer including blood group and tumor markers, which has high reference value for the diagnosis of gastric cancer patients, and the model can better distinguish early gastric cancer from healthy people.

## Introduction

Gastric cancer (GC) is an important cancer worldwide. According to the latest data of the International Agency for Research on Cancer (IARC), it ranks the 5th in incidence and the 4th in mortality globally 1. GC is the most common gastrointestinal tumor in China, of which the rate of early detection is low. Compared with 2015, GC ranked third in the number of new cases and mortality of common malignant tumors in 2020^2^, posing a serious threat to human health. Early GC can be treated by surgery, and the 5-year survival rate is more than 90%, while advanced GC can be treated by surgery, and the 5-year survival rate is less than 30% 3. The gold standard for screening early gastric cancer is gastroscopy. However, gastroscopy is not suitable for large-scale population screening and follow-up due to its detection rate of less than 10%, invasiveness, need of a large number of manpower and resources, low population acceptance and complications 4.

Tumor marker is a kind of substance synthesized and released by tumor itself. In the process of tumor occurrence and development, it is also accompanied by the modified expression of blood group antigen in tumor cells. Tumor markers have the advantages of non-invasive, easy specimen acquisition and low cost, which are suitable for dynamic monitoring. Some serum tumor markers (especially carcinoembryonic antigen [CEA], carbohydrate antigen [CA] 72-4, CA 19-9, and alfa fetoprotein [AFP]) have been reported to be elevated in some patients with gastric cancer and have been associated with the onset, progression, and recurrence of GC 5-7. However, the positive rate of tumor markers in advanced GC is only 20%-30%, and the positive rate in early GC is less than 10% 8. Although the sensitivity of combined serum tumor markers in the diagnosis of GC has been improved, it is still low 9. It is well known that the occurrence and development of gastric cancer are related to genetic factors, environmental factors and Helicobacter pylori infection 10-12. Blood group is one of the most stable genetic factors, among which ABO blood group is by far the most important blood group system 13. Since Aird et al. 14 first proposed that blood type A was associated with the occurrence of GC in 1953, a large number of studies on blood type and the incidence, clinicopathological characteristics and prognosis of GC have been carried out 15, 16. However, the relationship between ABO blood group and GC cannot be concluded with certainty due to conflicting findings at different studies 17-19. If the combined detection of blood group and tumor markers can improve the diagnostic efficiency of GC, the diagnostic timing, diagnostic cost and diagnostic accuracy of GC can be improved. Therefore, in this study, a diagnostic and predictive model for gastric cancer based on blood groups and tumor markers was constructed to predict the risk of gastric cancer in individuals with different blood groups and different levels of tumor markers.

## Materials and methods

### Patients

A total of 3650 GC patients who were treated in the Hospital of Digestive Diseases of the First Affiliated Hospital of Air Force Medical University from January 2015 to December 2019 were selected as the GC group, and 5822 healthy people who underwent physical examination in the Physical examination center of the hospital from January 2015 to March 2022 were selected as the control group. The 9472 subjects were divided into a training set and a validation set at a ratio of 7:3. There were 4091 healthy controls and 2552 gastric cancer patients in the training set. The validation set consisted of 1731 healthy controls and 1098 gastric cancer patients. All patients in the GC group underwent radical gastrectomy and were confirmed to be GC by surgical pathology, excluding those with previous malignant tumor history and incomplete data. The control group was the healthy population in the physical examination center, excluding those with malignant tumors and incomplete data. This retrospective study was reviewed by the Ethics Committee of the First Affiliated Hospital of Air Force Medical University and approval was obtained. All methods were carried out in accordance with relevant guidelines and regulations. All experimental protocols were approved by the Ethics Committee of the First Affiliated Hospital of Air Force Medical University. Due to the retrospective nature of the study, the informed consent was waived by the Ethics Committee of the First Affiliated Hospital of Air Force Medical University.

### Clinicopathological data

The baseline data of the GC included age, sex, ABO blood group, TNM stage, degree of differentiation, tumor location, lymph node metastasis, distant metastasis, and staging of GC. TNM staging was performed using American Joint Committee on Cancer (AJCC) Version 8. Tumor marker levels were measured 7 days before surgery. The baseline data of healthy controls included age, sex, blood type, and tumor marker levels. The serum tumor markers were determined after centrifugation of 3ml venous blood samples. Electrochemiluminescence (ECL) was used, and the instrument was the ECL analyzer and the matching kit provided by Roche Diagnostics (Germany). The reference values of CEA, CA199, CA125, AFP and CA724 were 5.0ng/ml, 27.0U/ml, 35 U/ml, 7.0ng/ml and 6.9U/ml, respectively.

### Statistical analysis

SPSS26.0(US, IBM SPSS) software was used for data analysis. Normal distribution data were expressed as mean ± standard deviation, and two independent samples were analyzed by t test. Non-normally distributed data were expressed as median and quartile, and comparison between groups was performed using the Mann-Whitney U test. Count data were expressed by [n (%)], and the comparison of rates was analyzed by χ2 test. In the training set, the binary Logistic regression method was used to establish a diagnostic model for gastric cancer. The ROC curve was made according to the P value of the prediction probability of gastric cancer, and the AUC, sensitivity and specificity were calculated to evaluate the diagnostic value of the diagnostic model for gastric cancer patients in the training set. The same method was used to determine the diagnostic value of the model for gastric cancer in the validation cohort. The ability of the diagnostic model to distinguish early gastric cancer was evaluated in the training set and validation set. The AUCs were compared using Delong test in MedCalc, version 20.0 (Solvu soft Corporation, American). A *P* value of less than 0.05 was considered to indicate statistical significance.

## Results

### Comparison of general clinical features

The study population was divided into training set and validation set according to 7:3. There were 4091 healthy controls and 2552 GC patients in the training set, 1731 healthy controls and 1098 GC patients in the validation set. There was no significant difference in the basic clinical characteristics between the training set and the validation set (*P* > 0.05, **Table 1**). Therefore, the study population selected in this study meets the experimental requirements and can be used for the construction and validation of the prediction model. The mean age of GC patients was 58±11 years (i.e., 21 to 89 years), and the mean age of healthy people was 50 ± 10 years (i.e., 18 to 87 years).

In both the training and validation sets, the level of tumor markers in the GC group was significantly higher than that in the healthy control group, and the difference was statistically significant (*P* < 0.05), as shown in **Table 2**. The constituent ratio of blood group distribution in GC group was: A > B > O > AB, while that in healthy control group was: B > O > A > AB. There were statistically significant differences in ABO distribution constituent ratios in the whole population (χ2=10.920, *P* =0.012, Table 3) and the training set (χ2=8.495, *P* =0.037, **Table 3**).

**Figure 1:**
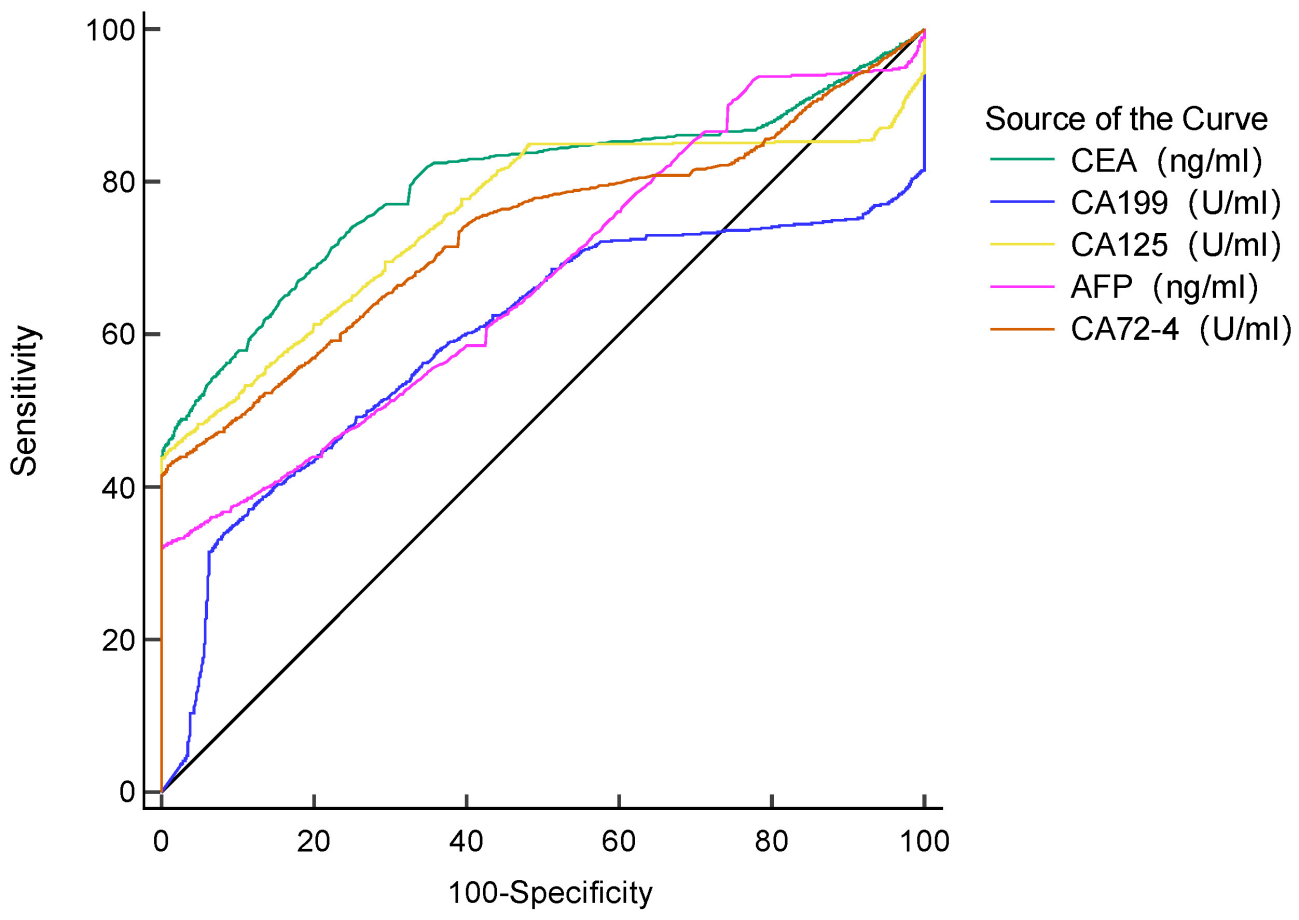
Diagnostic value of single tumor marker for GC patients.

The AUC of the five tumor markers ranged from 0.594-0.797, with a sensitivity of 32.22%-66.99%, which was at a low level (**Table 4**, **Figure 1**). The combined detection of tumor markers can improve its sensitivity and specificity. Compared with the combined detection of 5 tumor markers, the combination of type B and AB blood with 5 tumor markers can improve the diagnostic value of GC. The B blood group (AUC=0.936, 95%CI 0.927-0.945, *P* < 0.0001) combined with tumor markers detection was higher than that of tumor markers detection alone, and the difference was statistically significant. There was no significant difference in the AUC of AB blood (AUC=0.928, 95%CI 0.909-0.943, *P* =0.0566), but the *P* value was at the critical value of the test level (**Table 5**).

### Establishment and validation of a diagnostic model for gastric cancer based on binary Logistic regression

**Figure 2:**
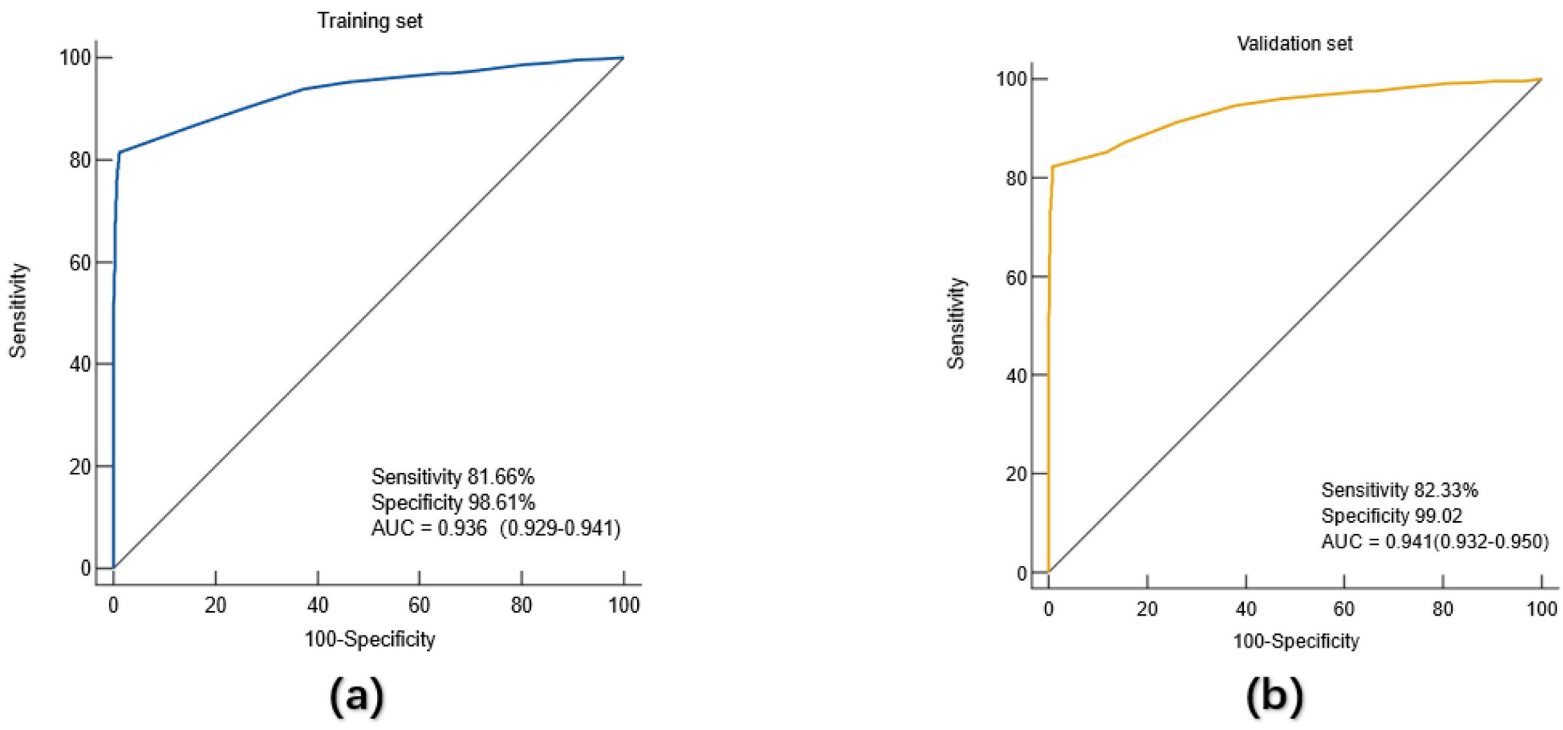
Diagnostic value of the diagnostic model for (a) training set and (b) validation set.

With gastric cancer as the dependent variable, binary Logistic regression analysis was used to screen indicators with diagnostic value for gastric cancer, and a regression equation was constructed. Finally, 8 indicators entered the equation, which were gender, age, CEA, CA199, CA125, AFP, CA724 and blood group. According to the formula PRE (*P*=GC) =1/ (1+EXP (-Logit (*P*))), the constant term and the regression coefficients of these eight indicators were put into the Logistic equation to obtain the corresponding predicted probability Logit (*P*) value for each subject. The diagnostic prediction model of gastric cancer obtained in this study is as follows: PRE (*P*=GC) =1/ (1+EXP (- (-2.490-0.929× sex +1.032× age +5.647×CEA+4.360×CA199+3.378×CA125+5.168×AFP+4.606×CA724-0.156× blood group B-0.278 ×O group Blood-0.098 ×AB blood))). The constant of the model is -2.490. In the index of gender, "male" is assigned a value of 1, and "female" is assigned a value of 2. "Age ≤45" was assigned a value of 0 and "age > 45" was assigned a value of 1. The negative value of CEA, CA199, CA125, AFP, CA724 was assigned 0, and the positive value was assigned 1. The index "blood type" was treated as A dummy variable, and "blood type A" was used as a reference variable. The predictive probability value of the model was used to draw the ROC curve, and the diagnostic value of the model for gastric cancer was evaluated. Results As shown in **Figure 2**, the AUC of the diagnostic model for gastric cancer in the training set was 0.936 (95%CI: 0.926-0.941), the sensitivity was 81.66%, the specificity was 98.61%, and the accuracy of the model for gastric cancer patients in the training set was 81.6%.

The diagnostic prediction model based on the training set was further validated by another population, namely the validation set. The ROC curve was drawn using the predicted probability *P* value. The results showed that in the validation set, the AUC of the diagnostic model for gastric cancer patients was 0.941 (95%CI: 0.932-0.950), the sensitivity was 82.33%, and the specificity was 99.02%. Similarly, further analysis showed that the accuracy of the model for gastric cancer patients in the validation group was 82.03%.

### To evaluate the diagnostic value of the diagnostic model in different stages of gastric cancer

**Figure 3:**
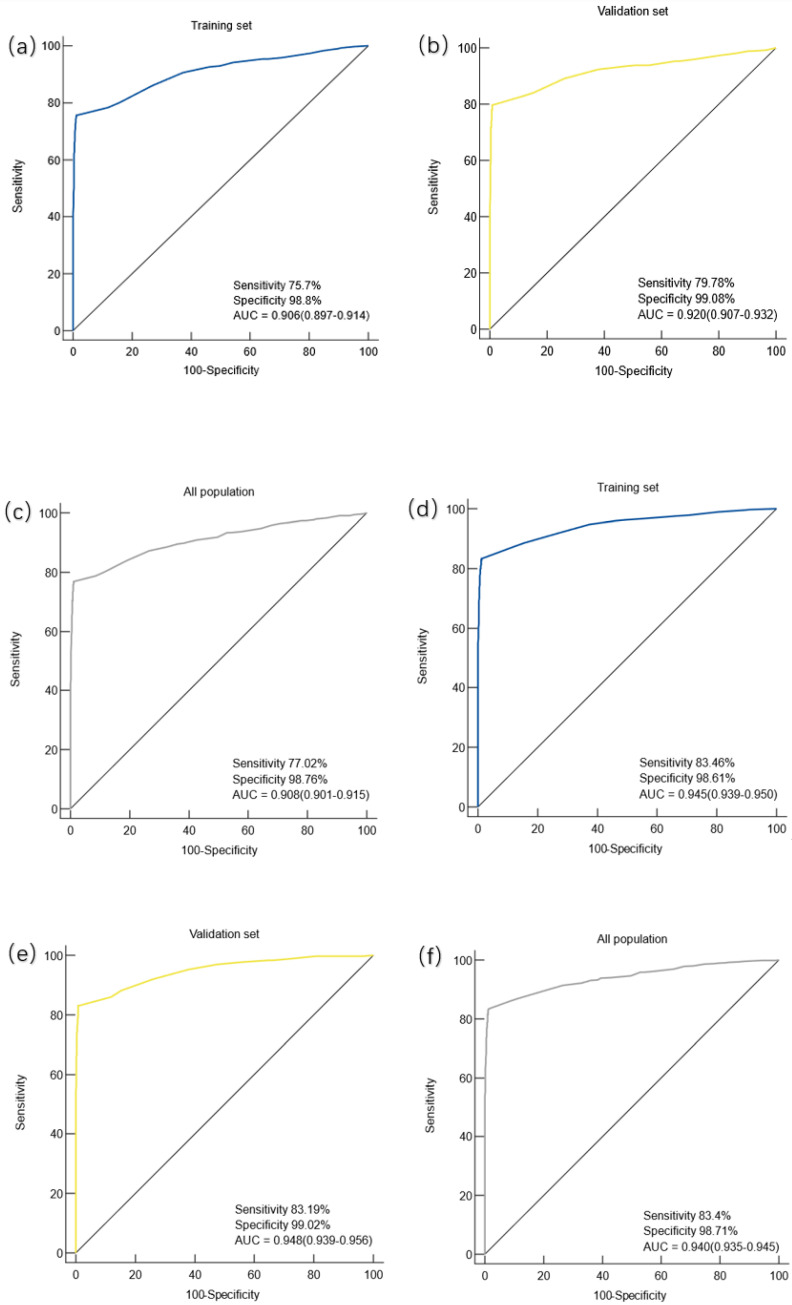
** Diagnostic value of the diagnostic model for early and advanced gastric cancer. Note:** (a) Diagnostic value of the diagnostic model for EGC in the training set; (b) the diagnostic value of the diagnostic model for EGC in the validation cohort; (c) diagnostic value of the diagnostic model for EGC in the overall population; (d) diagnostic value of the diagnostic model for AGC in the training set; (e) diagnostic value of the diagnostic model for AGC in the validation set; (f) diagnostic value of the diagnostic model for AGC in the overall population.

Gastric cancer patients were divided into early gastric cancer (EGC) and advanced gastric cancer (AGC) subgroups according to the American Joint Committee on Cancer (AJCC) 8th edition, EGC includes T1 grade tumors that are located in the mucosa (T1a) or reach the submucosa (T1b), regardless of the presence or absence of lymph node metastasis. The diagnostic efficacy of the constructed diagnostic model was evaluated in the patients of the two subgroups. The study found that in the training set, the AUC of the prediction model for the diagnosis of EGC patients was 0.906 (95%CI: 0.897-0.914), the sensitivity was 75.7%, and the specificity was 98.8%, and the difference was statistically significant (*P* < 0.0001) (**Figure 3.a**). In the validation cohort, the AUC of the prediction model for EGC patients was 0.920 (95%CI: 0.907-0.932), with a sensitivity of 79.78% and a specificity of 99.08%, and the difference was statistically significant (*P* < 0.0001) (**Figure 3.b**). In the overall population, the AUC of the prediction model for patients with EGC was 0.0.908 (95%CI: 0.0.901-0.915), the sensitivity was 77.02%, and the specificity was 98.76%, and the difference was statistically significant (*P* < 0.0001) (**Figure 3.c**).

In the training set, the AUC of the prediction model for the diagnosis of AGC was 0.945(95%CI: 0.939-0.950), the sensitivity was 83.46%, and the specificity was 98.61%, and the difference was statistically significant (*P* < 0.0001) (**Figure 3.d**). In the validation cohort, the AUC of the prediction model for AGC was 0.948 (95%CI: 0.939-0.956), the sensitivity was 83.19%, and the specificity was 99.02%, with a statistically significant difference (*P* < 0.0001) (**Figure 3.e**). In the overall population, the AUC of the prediction model for patients with AGC was 0.940 (95%CI: 0.935-0.945), the sensitivity was 83.4%, and the specificity was 98.71%, and the difference was statistically significant (*P* < 0.0001) (**Figure 3.f**).

## Discussion

So far, a large number of studies have explored the value of tumor markers on the incidence of gastric cancer 20, 21, clinicopathological features and prognosis 22-24. The sensitivity of single tumor markers in detecting gastric cancer is insufficient. Studies have explored the diagnostic value of CEA, CA724, CA199 and CA125 for gastric cancer, and the results suggest that the sensitivity of these tumor markers in diagnosing gastric cancer is between 20% and 40%, and the sensitivity of combining these four tumor markers is only 60.9%^25^.In this study, it was found that the sensitivity of single tumor markers in the diagnosis of gastric cancer was 32%-67%, and the sensitivity increased to 91.7% after combined detection, which was somewhat inconsistent with previous reports. It may be that this study only included patients with gastric cancer and healthy people, but not those with precancerous lesions, resulting in increased sensitivity.

This study found that there were differences in ABO blood group distribution between the gastric cancer group and the healthy control group. The constituent ratio of blood group distribution in GC group was A > B > O > AB, and the healthy control group was B > O > A > AB, and the difference in ABO distribution composition ratio was statistically significant. Two studies on the correlation between ABO blood group and gastric cancer in Jilin 26 and Shanghai 27 found that the distribution composition of ABO blood group in gastric cancer patients and healthy people was slightly different from that in this study, but both suggested that blood group A accounted for the highest proportion of gastric cancer patients.

There are few studies on the diagnostic value of ABO blood group combined with tumor markers for gastric cancer. In this study, binary logistic regression method was used to construct a gastric cancer diagnosis and prediction model containing 8 indicators (gender, age, CEA, CA199, CA125, AFP, CA724 and blood group) in the training set: PRE (*P*=GC) =1/ (1+EXP (- (-2.490-0.929× sex +1.032× age +5.647×CEA+4.360×CA199+3.378×CA125+5.168×AFP+4.606×CA724-0.156× blood group B-0.278 ×O Blood type -0.098×AB)), and the ROC curve of the model was drawn by the prediction probability *P* value to evaluate the diagnostic value of the model for gastric cancer patients in the training set, and then the model was validated in the validation set. The results showed that in the training set, the AUC of the model for gastric cancer diagnosis was 0.936, the sensitivity was 81.66%, the specificity was 98.61%, and the judgment accuracy was 81.6%. In the validation set, the AUC of the model for gastric cancer diagnosis was 0.941, the sensitivity was 82.33%, the specificity was 99.02%, and the accuracy was 82.03%. All these suggest that this model has a good diagnostic value for gastric cancer.

In this study, gastric cancer was further divided into EGC and AGC to evaluate the diagnostic value of the diagnostic model for EGC and AGC respectively. The results showed that the AUC of the prediction model for EGC in the training set, validation set and overall population were 0.906, 0.920 and 0.908, respectively. It is suggested that the diagnostic value of the model for EGC is high whether in the training set, validation set or the overall population, indicating that the diagnostic model obtained in this study can better distinguish EGC patients from the healthy population. Therefore, it is recommended to use the model in high-risk populations of EGC and to consider whether to undergo further examination, such as electronic gastroscopy, based on the results of the model.

Our current study has several limitations. Firstly, due to the limitation of conditions, ABO blood group and tumor marker level information of patients with GC precancerous diseases could not be obtained, and patients with GC precancerous diseases were not included, so the objectivity of the results was slightly weak. Secondly, this study is a retrospective study, and prospective study subjects are needed to further verify the diagnostic value of the diagnostic prediction model for GC.

In conclusion, we proposed and validated a GC prediction model including blood group and tumor markers, which showed excellent performance in the diagnosis and accuracy of GC. In addition, the model also shows good diagnostic value in predicting EGC, which helps clinicians to provide new ideas for the clinical diagnosis of gastric cancer.

## Figures and Tables

**Table 1:** The clinicopathological characteristics of the study population, number (%)

Characteristics	Training set (N=6643)	Validation set (N=2829)	*t*/χ^2^	*P*
**Healthy controls**	4091	1731		
Age, y				0.813
Mean± standard deviation	50±10	50±10		
Gender			1.673	0.196
Male	2134 (52.2)	935 (54.0)		
Female	1957 (47.8)	796 (46.0)		
ABO blood group			1.437	0.697
A	1178 (28.8)	519 (30.0)		
B	1259 (30.8)	525 (30.3)		
O	1224 (29.9)	519 (30.0)		
AB	430 (10.5)	168 (9.7)		
**Patients with GC**	2552	1098		
Age, y				0.915
Mean± standard deviation	58±11	58±11		
Gender			0.176	0.675
Male	1896 (74.3)	823 (75.0)		
Female	656 (25.7)	275 (25.0)		
ABO blood group			1.017	0.797
A	818 (32.1)	358 (32.6)		
B	769 (30.1)	320 (29.1)		
O	714 (28.0)	302 (27.5)		
AB	251 (9.8)	118 (10.7)		
TNM stage			3.450	0.327
I	716 (28.1)	335 (30.5)		
II	510 (20.0)	203 (18.5)		
III	1202 (47.1)	515 (46.9)		
IV	124 (4.9)	45 (4.1)		
Differentiation			7.001	0.136
Well differentiation	62 (2.4)	43 (3.9)		
Well and middle differentiation	57 (2.2)	20 (1.8)		
Middle differentiation	509 (19.9)	222 (20.2)		
Middle and low differentiation	316 (12.4)	127 (11.6)		
Low differentiation	1608 (63.0)	686 (62.5)		
Tumor site			2.079	0.556
Gastric fundus and cardia	282 (11.1)	125 (11.4)		
Gastric body Antrum	838 (32.8) 1283 (50.3)	349 (31.8) 547 (49.8)		
Whole stomach	149 (5.8)	77 (7.0)		
Lymphatic metastasis			2.343	0.126
N0	1568 (61.4)	645 (58.7)		
N1-3	984 (38.6)	453 (41.3)		
Remote metastasis			1.006	0.316
M0	124 (4.9)	45 (4.1)		
M1	2428 (95.1)	1053 (95.9)		
Stage			1.183	0.277
Early stage	601 (23.6)	277 (25.2)		
Advanced stage	1951 (76.4)	821 (74.8)		

**Table 2:** Differences in tumor marker levels between GC group and healthy controls, median (IQR)

	Patients with GC	Healthy subjects	Z	*P*
CEA (ng/ml)				
Training set	3.875 (2.250,105.000)*	1.620 (1.080,2.330)	-40.048	<0.001
Validation set	4.280 (2.378,106.250)	1.600 (1.030,2.360)	-27.908	<0.001
CA199 (U/ml)				
Training set	6.290 (1.733,20.075)	9.050 (6.120,13.590)	-13.194	<0.001
Validation set	6.305 (1.850,15.650)	8.620 (5.890,12.890)	-7.871	<0.001
CA125 (U/ml)				
Training set	5.505 (1.560,8.868)	10.180 (7.680,13.590)	-35.102	<0.001
Validation set	5.790 (1.578,8.750)	10.140 (7.630,13.660)	-22.221	<0.001
AFP (ng/ml)				
Training set	3.720 (2.590,27.095)	2.880 (2.100,3.910)	-23.428	<0.001
Validation set	3.730 (2.520,17.550)	2.830 (2.050,3.800)	-15.612	<0.001
CA72-4 (U/ml)				
Training set	4.580 (2.022,47.600)	1.610 (0.980,2.960)	-32.623	<0.001
Validation set	4.620 (2.000,44.615)	1.680 (1.020,3.040)	-20.392	<0.001

**Note:** *Values are expressed as medians and interquartile ranges.

**Table 3:** Distribution of ABO blood groups

	Patients with GC (%)	Healthy subjects (%)	χ^2^	*P*
A				
Training set	818 (32.10)	1178 (28.80)	8.495	0.037
Validation set	358 (32.60)	519 (30.00)	3.949	0.267
B				
Training set	769 (30.10)	1259 (30.80)		
Validation set	320 (29.10)	525 (30.30)		
O				
Training set	714 (28.00)	1224 (29.90)		
Validation set	302 (27.50)	519 (30.00)		
AB				
Training set	251 (9.80)	430 (10.50)		
Validation set	118 (10.70)	168 (9.70)		

**Table 4:** Diagnostic value of single tumor marker in patients with GC

TM	AUC	95%CI	TPR (%)	TNR (%)	Youden index
CEA	0.797	0.789-0.806	66.99	82.05	0.4904
CA199	0.594	0.584-0.604	34.05	91.64	0.2569
CA125	0.753	0.744-0.762	44.68	99.07	0.4376
AFP	0.672	0.662-0.681	32.22	99.71	0.3193
CA724	0.734	0.725-0.743	42.79	99.02	0.4182

**Note:** TM: tumor maker; CI: confidence interval; TPR: true positive rate; TNR: true negative rate

**Table 5:** Combined diagnosis compared with stratified combined diagnosis

TM	AUC	95%CI	TPR (%)	TNR (%)	Youden index	Z	*P*
TM	0.917	0.912-0.923	78.82	97.39	0.7621	116.750	<0.0001
A+TM	0.908	0.897-0.919	80.53	95.99	0.7652	60.185	<0.0001
B+TM	0.936	0.927-0.945	80.17	95.85	0.7602	81.086	<0.0001
O+TM	0.911	0.900-0.921	79.92	96.90	0.7682	57.473	<0.0001
AB+TM	0.928	0.909-0.943	78.32	98.33	0.7665	42.531	<0.0001
